# Features of the Gas-Permeable Crystalline Phase of Poly-2,6-dimethylphenylene Oxide

**DOI:** 10.3390/polym14010120

**Published:** 2021-12-29

**Authors:** Alexander Yu Alentiev, Ivan S. Levin, Nikolay A. Belov, Roman Yu Nikiforov, Sergey V. Chirkov, Denis A. Bezgin, Victoria E. Ryzhikh, Julia V. Kostina, Victor P. Shantarovich, Leonid Yu Grunin

**Affiliations:** 1 A.V. Topchiev Institute of Petrochemical Synthesis RAS, 119991 Moscow, Russia; levin@ips.ac.ru (I.S.L.); belov@ips.ac.ru (N.A.B.); nru@ips.ac.ru (R.Y.N.); sm1th@ips.ac.ru (S.V.C.); bezgin@ips.ac.ru (D.A.B.); kochenkova@ips.ac.ru (V.E.R.); julia@ips.ac.ru (J.V.K.); 2 N.N. Semenov Federal Research Center For Chemical Physics RAS, 119991 Moscow, Russia; shant@center.chph.ras.ru; 3 Department of Physics, Volga State University of Technology, 424000 Yoshkar-Ola, Russia; GruninLY@volgatech.net

**Keywords:** poly-2,6-dimethylphenylene oxide, gas permeability, free volume, semi-crystalline polymers, gas sorption, organic molecular sieves

## Abstract

Poly-2,6-dimethylphenylene oxide (PPO) film samples with varying degrees of crystallinity (from 0 to 69%) were obtained by means of different techniques. The films were studied by various physicochemical methods (Fourier-transform infrared spectroscopy, positron annihilation lifetime spectroscopy, X-ray diffraction, and ^1^H nuclear magnetic resonance relaxation). Solubility coefficients of gases in the PPO samples were measured via sorption isotherms of gases by volumetric technique with chromatographic detection. The apparent activation energy of permeation and the activation energy of diffusion of all gases were estimated based on temperature dependences of gas permeability and diffusivity for amorphous and semi-crystalline PPO in the range of 20–50 °C. The peculiarities of free volume, density, and thermal properties of gas transport confirm the nanoporosity of the gas-permeable crystalline phase of PPO. So, the PPO can be included in the group of organic molecular sieves.

## 1. Introduction

**Figure 1:**
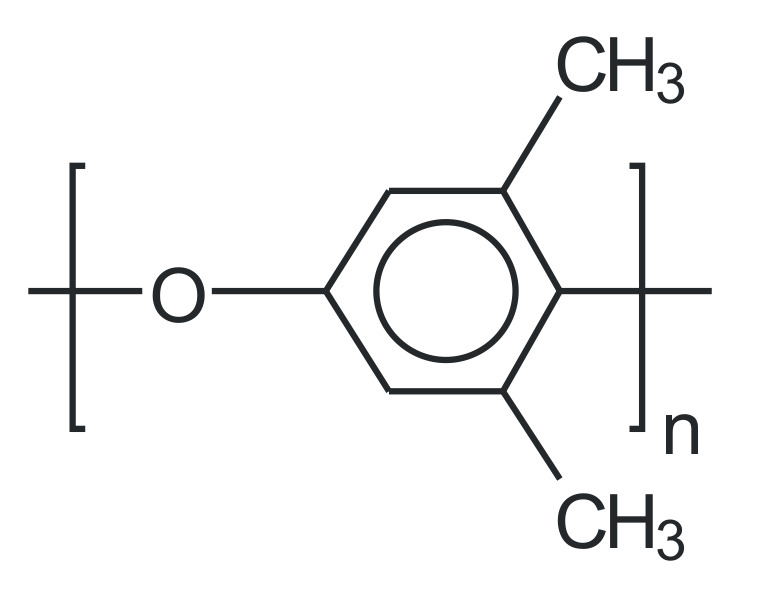
Chemical structure of PPO.

Membrane processes are widely used for separation of mixtures of non-condensable gases [1]. The common membrane materials of thin selective layers for these applications are glassy polymers of different chemical natures, whereas semi-crystalline polymers were not found to be significant spread because of the low permeability of the crystalline phase (orders of magnitude lower than the amorphous one). However, there are several exceptions when the crystalline phase of the polymer is loosely packed and permeable to gases: poly(4-methyl pentene-1) [2,3] and poly-2,6-dimethylphenylene oxide (PPO, Figure 1) [4,5,6].

Numerous publications have been devoted in the investigation of internal structure and macroscopic parameters of PPO [4,5,6,7,8,9,10,11,12,13,14]. In our previous research, it was demonstrated that the density of the crystalline phase is significantly lower than that of the amorphous one, and the permeability of the crystalline phase is, respectively, significantly higher [5]. Additionally, the high gas permeability of semi-crystalline PPO films prepared from chloroform solution is mainly due to the high permeability of the β-modification of the crystalline phase. At the same time, the structure of semi-crystalline PPO films was proposed to be considered as the dispersion of nanocrystallites (up to 7 nm in size) in an amorphous matrix [5,15]. The current investigation aims to continue the study of the behavior of semi-crystalline phase of PPO by means of the various physicochemical methods. Namely, the PPO samples formed from chloroform solutions with a different degree of crystallinity are tested by X-ray diffraction, IR spectroscopy, ^1^H NMR relaxation, PALS, and the gas sorption technique. In addition, the thermal parameters of gas transport (the apparent activation energy of gas permeation, the activation energy of diffusion, and the heat of sorption) were estimated based on temperature dependencies of the corresponding gas transport properties. This complex approach should supplement and expand an unambiguous picture of the PPO structure developed by other scientific groups [6,7,8,9,10,11,12,13,14].

## 2. Materials and Methods

In the present work, the same PPO powder samples were used for PPO films preparation, as in [5]; same designations were used: PPO-1 (M_w_ = 636 kDa) synthesized in the 1990s in the Institute of Chemical Technology (Novosibirsk, Russia), and PPO-2 (M_w_ = 355 kDa) and PPO-4 (M_w_ = 610 kDa) samples provided by Research and Manufacturing Association (RMA) Geliymash (Moscow, Russia). These PPO samples were chosen because PPO-2 has the lowest degree of crystallinity, PPO-1 has the medium degree of crystallinity, and PPO-4 has the highest degree of crystallinity [5]. Chloroform, reagent grade, was used as the solvent.

### 2.1. Film Preparation

All PPO films (PPO-1, PPO-2, and PPO-4), with a thickness of 40–50 microns, were prepared by casting the solution in chloroform (5 wt%) on a cellophane support and drying at room temperature for 2–3 days with subsequent evacuation to a constant mass. An amorphous film of PPO-2 (PPO-2/50 °C) was formed from a solution in chloroform (5 wt%) on a cellophane support and dried on a heated table at 50 °C.

### 2.2. Film Characterization

Polymer films densities (ρ) were determined at room temperature (24 ± 2 °C) by hydrostatic weighing in isopropanol. Fractional free volume (FFV) was calculated using Bondi method: FFV = 1 − 1.3 V_w_/V_sp_, where V_w_ is Van der Waals volume of repeat unit, V_sp_ = M/ρ is a specific volume of polymer, and M is polymer repeat unit molecular weight.

The permeability and diffusion coefficients of H_2_, He, N_2_, O_2_, CO_2_, and CH_4_ gases for the obtained free films were measured by an integral barometric method on the thermostated MKS Barotron installation; LabVIEW-based software was used to control the experiment. The experiments were carried out at various temperatures from room temperature to 50 °C, and at upstream pressure in the range of 0.7–0.9 atm. The downstream pressure was maintained at the level of ~10^−3^ mmHg; therefore, in the conditions of the experiment, the back diffusion of the penetrant was neglected. Permeability coefficient *P* and diffusion coefficient *D* were measured using the curve of gas permeation through the polymer film into a calibrated volume. *P* was determined by the slope tangent of the linear dependence of the flow through a film after the steady-state mass transfer was reached. *D* was determined by the Daynes–Barrer time lag (*θ*, s) method: *D* = *l^2^/6 θ*, where l is the film thickness. Due to the small time lags (*θ* < 1 s), helium and hydrogen diffusion coefficients were not determined. The solubility coefficients *S* were calculated as *P*/*D*. Ideal separation selectivities (*α* = *P_i_*/*P_j_*) and diffusion selectivities (*α^D^* = *D_i_*/*D_j_*) were found for different *i* and *j* gas pairs using the obtained data. The experimental measurement error was 5% and 10% for *P* and *D*, respectively. The calculation errors were 15%, 10%, and 20% for *S*, *α*, and *α^D^*, respectively. The temperature dependences of the *P*, *D*, and *S* values were determined at 5–6 different temperatures in the range of 22–50 °C. These dependences were processed within the framework of the activated state theory: ln*P* = ln*P*_0_ − *E_P_*/*RT* and ln*D* = ln*D*_0_ − *E_D_*/*RT*, and the Kirchhoff equation: ln*S* = ln*S*_0_ − ∆*H_S_*/RT in Arrhenius coordinates from the inverse temperature.

X-ray diffraction (XRD) studies of PPO films were performed with Rigaku diffractometer (Tokyo, Japan). Diffractograms were obtained using an X-ray source with a rotating copper anode Rotaflex RU-200 and the operating mode of 50 kV–100 mA. The source was equipped with a horizontal wide-angle Rigaku D/Max-RC goniometer and a secondary graphite monochromator (the wavelength λ of the monochromatic radiation was 1.542 angstroms). The range of diffraction angles measurement was 3–40 degrees for 2*θ*. The measurement was carried out in a continuous scanning mode at a speed of 2 degrees/min and a step of 0.04 degrees. *θ*–2*θ* scanning was performed using Bragg–Brentano scheme. Film samples were fixed on aluminum frames, and scanning was performed in the reflection mode. With the X-ray wavelength used in the reflection mode, the beam scanned the entire depth of the films. Information on the degree of crystallinity of PPO powders was reported earlier in [5].

The resulting diffractograms were processed using the Fityk program [16]: after subtracting the background, the diffractogram was approximated by the sum of seven Gaussian peaks, two of which (with angular positions of about 14 and 23 degrees), according to [5], were assigned to amorphous phase when calculating the degree of crystallinity. The degree of crystallinity *C_I_* of samples was calculated using the Ruland method [17] by the formula $C_{l}=\frac{A_{cr}}{A_{sum}}$*,* where *A_cr_* is the sum of the integral intensities (areas) of the peaks corresponding to the crystalline phase and *A_sum_* is the total peak area that approximated the diffractogram. The size of the crystallites *D_hkl_* along the *hkl* crystallographic direction for each peak was estimated using the Scherrer equation: $D_{hkl}=\frac{K\times \lambda }{FWHM\times cos\theta_{hkl}}$, where *λ* is the X-ray wavelength, *K* = 0.94 is the Scherrer constant for spherical particles, and FMHW is the half-height width of the peak with an angular position of 2*θ_hkl_*.

Studies by positron annihilation lifetime spectroscopy [18,19] were carried out with EG and G ORTEC (USA) spectrometer with the ^44^Ti (0.5 MBq) radioactive source of positrons and the ORTEC-TRUMP-PCI-2K buffer device at ambient conditions. The measurement results (positron lifetimes and intensities) were determined as an average based on the results of several experiments. The well-known PATFIT [19] program was used for mathematical processing. The four-component fitting (with two long *ortho*-positronium lifetime components τ_3_, I_3_ and τ_4_, I_4_) gave a completely satisfactory description, similarly to the LT 9.0 program [19]. The lifetime of *ortho*-positronium in the studied substance was used to determine the effective size of nanopores (R_3_, R_4_) using the Tao–Eldrup formula [19], with pores represented as spherically symmetric potential wells with infinitely high walls.

Sorption measurements were carried out by the volumetric method with chromatographic detection [20] using the original installation. A metal tube with an inner diameter of 2 mm and a length of 90 mm was used as a sorption cell (loop), into which a polymer film of a known mass and cut into strips was placed. Preparation of the sorption volume was carried out by evacuating the loop. Then, the sorption volume with a polymer film was saturated with a gas at a certain pressure. After complete saturation of the sample, the amount of gas in the cell was detected using a thermal conductivity detector of the Crystallux-4000 chromatograph (Yoshkar-Ola, Russia). The amount of gas sorbed in the polymer film was determined based on the calculations described in [20]. The sorption isotherms of nitrogen and oxygen, carbon dioxide, and methane were measured at 35 °C in the range of 0–1.2 and 0–6 atm, respectively. Based on the obtained data, sorption isotherms were plotted and the solubility coefficients were calculated by the slope of the initial part of the isotherm (0.1–1.2 atm for nitrogen and oxygen, 0.1–2 atm for carbon dioxide and methane).

NMR relaxation (Time-Domain NMR, TD-NMR) measurements were done with the Resonance Systems Spin Track NMR analyzer (Kirchheim unter Teck, Germany) [21] operating at 18 MHz for ^1^H nuclei and equipped with variable sample temperature controller. PPO films were densely put into test tubes of 10 mm outer diameter.

Spin-lattice relaxation times were estimated with the “Saturation-Recovery” pulse sequence that contained 24 points for each acquired curve with 8 scans per every point. Values of T1 were calculated automatically by the “Relax8 Spin Track” software using single exponential model. The experiments were carried out both for PPO powders (semi-crystalline and amorphous [5]) and for films obtained in this work.

All measurements were done at three temperatures: 30, 50, and 88 °C.

The infrared spectra of the attenuated total reflectance (ATR-FTIR spectra) were recorded with the IFS 66 v/s (Bruker) FTIR spectrometer (Ettlingen, Germany) using ATR objective lenses (Ge, ZnSe crystals, resolution is 2 cm^−1^) at ambient conditions, in the range of 4000–600 cm^−1^. The spectra were processed using OPUS (Bruker) software.

## 3. Results and Discussion

### 3.1. XRD of the PPO Films

**Figure 2:**
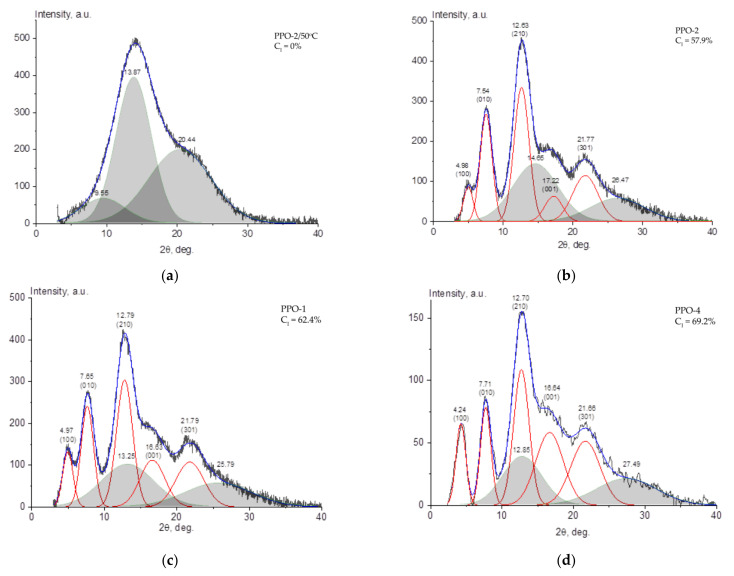
Diffractograms of PPO film samples with peak deconvolution. Grey peaks represent an amorphous phase. Red line peaks correspond to the peaks crystalline phase, blue line is a sum of the red peaks. Values over the peaks of crystalline phase are d-spacings and Miller indices (in parentheses). (**a**) PPO-2/50 °C film (C_I_ = 0%); (**b**) PPO-2 film (C_I_ = 57.9%); (**c**) PPO-1 film (C_I_ = 62.4%); and (**d**) PPO-4 film (C_I_ = 69.2%).

Diffractograms of PPO film samples were processed similarly to [5]; the results are shown in Figure 2.

In the diffractogram of an amorphous PPO-2/50 °C film, three broad peaks of the amorphous halo were observed: two with angular positions of about 13.5 and 20 degrees (these were also observed for the amorphous powder [5]) and another peak with an angular position of 8.5 degrees. As it was suggested in [5], this additional peak indicates the presence of crystalline phase nuclei in the film. Further, when deconvolution of diffractograms of semi-crystalline films was performed, this peak was not taken into account.

Based on the X-ray diffraction pattern, it can be concluded that in PPO-2 and PPO-1 nanocrystalline phase is present with peak positions characteristic of the β-modification of the crystalline phase in the nanocrystalline films of PPO described in [12,13]. However, it seems that in the PPO-4 sample, in addition to the β-modification, a nanocrystalline α-modification [12,13] is also present with its characteristic ratio of peak intensities and peak positions at 4.2 and 7.7 degrees. Nevertheless, this fact was not taken into account when calculating the degree of crystallinity since it was deemed incorrect to calculate the phase ratio due to the overlap of the corresponding diffraction peaks.

The values of the Miller indices *hkl* are provided in Table 1 in accordance with the data given in [11]; the angular positions of the “crystalline” diffraction maxima 2*θ*, the d-spacings calculated for them, the relative intensities of the diffraction maxima, the crystallite sizes calculated using the Scherrer formula, and the calculated degrees of crystallinity C_I_ for all of the samples studied are provided for all diffraction maxima.

According to [11], the nanocrystalline β-modification of the crystalline phase is characterized by an orthorhombic unit cell with periods of 17–19 Å in the *a* direction, 11–11.6 Å in the *b* direction, and 5.1–5.4 Å in the *c* direction. As one can see from Table 1, with the increase in the degree of crystallinity of the films, the calculated volume of the crystal unit cell (*a* × *b* × *c*) also increases, but the sizes of the crystallites along various crystallographic directions practically do not change. This fact may also indirectly indicate the presence of a nanocrystalline α-modification of the crystalline phase in the PPO-4 sample, which, according to [11], forms a continuous channel of a larger volume than the nanoporous β-modification.

### 3.2. ATR-FTIR Spectroscopy

The thickness of the films used in the experiments described above did not allow for the use of IR transmission spectroscopy to register spectral features of amorphous and crystalline phases, including various modifications of the latter. To analyze the structure of the surface layer of the films, two ATR crystals were used, differing in the refractive index and, hence, the depth of penetration of radiation into the sample.

The authors of [11,12] confirm the presence of the amorphous phase, as well as the α- and β-modifications of the nanocrystalline phase, by individual sets of absorption bands in the IR spectra of PPO films. The absorption band with a maximum at 830 cm^−1^ and the absence of an absorption band at 777 cm^−1^ are interpreted as signs of an amorphous state of PPO. According to the literature data, the combination of absorption bands with maxima at 828, 773, and 414 cm^−1^ corresponds to α-modification of the nanocrystalline phase, and bands with maxima at 825, 777, and 418 cm^−1^ indicate the presence of β-modification.

**Figure 3:**
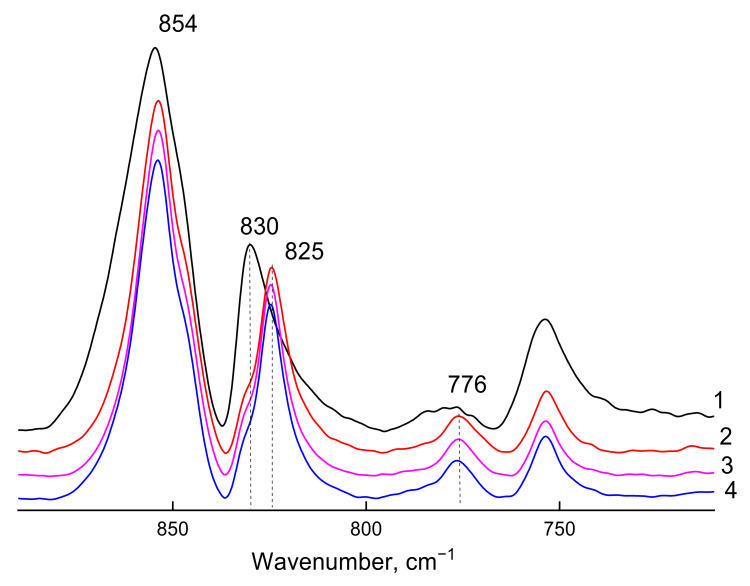
Fragments of ATR-FTIR spectra of PPO-2/50 °C (curve 1), PPO-1 (curve 2), PPO-4 (curve 3), and PPO-2 (curve 4) films in the vibrations absorption region characterizing the amorphous and crystalline phases (ZnSe crystal).

Analysis of the films ATR-FTIR spectra recorded using ZnSe crystal (2 µm depth of radiation penetration into the sample) has shown different structures of the PPO-1, -2, -4, and PPO-2/50 °C samples only as the difference between the amorphous state and β the modification of nanocrystalline phase (Figure 3).

PPO-2/50 °C film is characterized mainly by an amorphous structure (curve 1, 2); however, the asymmetry of the 830 cm^−1^ absorption band in long-wavelength part of the spectrum indicates the presence of ordered regions in the film (more precisely, on its surface). Taking into account the depth of radiation penetration into the sample when using the ZnSe ATR crystal (2 μm), it is possible to assume different morphology of the near-surface layers along the gradient of the film thickness.

There are signs of the chain ordering, i.e., crystalline phase presence, observed in the ATR-FTIR spectra of the PPO-1, PPO-2, and PPO-4 films: the absorption band maximum of deformational oscillations shifted to the long-wavelength region (to 824 cm^−1^), and the absorption band maximum at 776 cm^−1^ (characteristic of the β-modification of the crystalline phase according to [11,12]) appeared. The presence of the band shoulder at 830 cm^−1^ in the ATR-FTIR spectra of the films corresponds to the presence of disordered (amorphous) regions in the samples. No spectral signs of α-modification of the crystalline phase were observed in the PPO-4 spectrum. Considering that, according to the XRD data, both α- and β-modifications are observed in the PPO-4 film, it is possible to assume a gradient in the morphology of the crystalline phase in the near-surface layers of the films.

**Figure 4:**
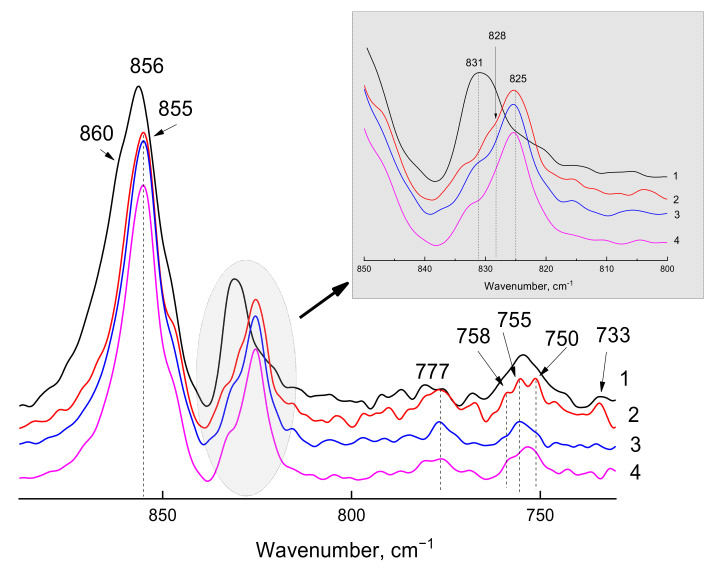
Fragments of ATR-FTIR spectra of PPO-2/50 °C (curve 1), PPO-1 (curve 2), PPO-4 (curve 3), and PPO-2 (curve 4) films in the vibrations absorption region characterizing the amorphous, α-, and β-modifications of crystalline phase obtained using Ge crystal. 800–850 cm^−1^ region is presented separately in grey box.

To answer this question, we analyzed the ATR-FTIR spectra recorded using the Ge ATR crystal (the depth of radiation penetration into the sample is 0.66 μm, Figure 4).

Interestingly, the position of the absorption band maximum corresponding to the amorphous phase in the ATR-FTIR spectrum recorded on the Ge crystal for the PPO-2/50 °C film exactly coincides with the literature data obtained for PPO films from the IR transmission spectra [11,12]. A slight shift of this maximum to the long-wavelength region was observed in the ATR-FTIR spectrum obtained using ZnSe. However, taking into account the extremely small difference in the position of the absorption bands identified by the authors [11,12] as corresponding to the amorphous phase and α- and β-modifications of the crystalline phase (831, 828, and 825 cm^−1^, respectively), it is the thin near-surface layer of the PPO film that is most spectrally sensitive to changes in the packing of polymer chains but deeper than 1 µm such differences are negligible. The second and the third spectral regions described in [11,12] for the IR transmission spectra cannot be used to identify phases by the ATR-FTIR spectra due to a very low intensity of the absorption bands in the 770–777 cm^−1^ region, and to 440 cm^−1^ region being out of the spectral range of ATR crystals used in the present work.

Unlike the PPO-2/50 °C film, which is mainly characterized by an amorphous structure, signs of the ordering, i.e., crystalline phase, were observed in the ATR-FTIR spectra obtained using the ATR-Ge crystal for PPO-1, PPO-2, and PPO-4: the absorption band maximum of deformational oscillations, which is observed at 831 cm^−1^ for the amorphous phase, shifted to the long-wavelength region (to 825 cm^−1^), typical of β-modification of the crystalline phase according to [11,12]. According to the same data, the crystal phase of the PPO-1 film (curve 2, Figure 4) consists of α- and β-modifications (absorption band maxima at 733 and 777 cm^−1^, respectively). If one refers only to the data of [11,12], then the absence of an absorption band at 733 cm^−1^ indicates the absence of α-modification of the crystalline phase in the PPO-2 and PPO-4 films. However, according to the XRD data, the calculated volume of the PPO-2 crystal unit cell is significantly less than that of the PPO-4 film (Table 1), i.e., the nanocrystalline structure of PPO-2 films differs from that of the PPO-4 film. In the ATR-FTIR spectrum, such differences are observed in the region of skeletal vibrations (750 cm^−1^): wide envelope curve for amorphous PPO-2/50 °C film, three maxima (at 758, 755, and 750 cm^−1^) for the PPO-1 film, and an inverse relationship in the ratio of the optical density values A_755_/A_750_ for the PPO-2 and PPO-4 films (1.26 and 0.68, respectively). It is reasonable to believe that the absorption band in the region of 750 cm^−1^ characterizes the features of the nanocrystalline structure of PPO in a thin near-surface layer.

Given that PPO-1 has both modifications of the nanocrystalline phase (α- and β-), it is logical to attribute shorter wavelength absorption band (758 cm^−1^) to the more common β-modification, the 755 cm^−1^ maximum is attributed to the amorphous structure, while the 750 cm^−1^ band can possibly be due to other modification of the nanocrystalline phase.

### 3.3. Density and Free Volume of the PPO Films

The density of the PPO-2/50 °C sample (Table 2) is close to the literature data for PPO [4,22,23,24,25] (1.067–1.070 g/cm^3^) and slightly lower than the data of [5] for amorphous PPO (1.079 g/cm^3^). The PPO-1, PPO-2, and PPO-4 semi-crystalline samples densities (Table 2) are slightly less than those published in [5] (1.051, 1.066, and 1.036 g/cm^3^, respectively). Similar to [5], the degree of crystallinity and free volume of PPO samples increase with the decrease in density.

The free volume of PPO film samples was also investigated by positron annihilation lifetime spectroscopy. The data are presented in Table 3.

A four-component PALS spectrum was not observed for an amorphous PPO-2/50 °C sample, and the obtained data were typical for “conventional” amorphous polymers. The fourth component appeared in the positron annihilation lifetime spectra of semi-crystalline PPO-1, PPO-2, and PPO-4 samples. The lifetimes of the fourth component of the spectrum and the corresponding “hole” sizes for PPO-1 and PPO-4 were equal within the measurement error, while they were slightly smaller for PPO-2 (Table 3). At the same time, the intensity of the fourth component of the spectrum increased with degree of crystallinity. In essence, it can be assumed that the appearance of the crystalline phase led to formation of 5.0–5.2 Å “holes”, which characterized the loosely packed crystalline phase; the concentration of the “holes” increased with degree of crystallinity and volume fraction of the crystalline phase. At the same time, the lifetimes of the third component of the spectrum and the corresponding smaller “hole” sizes, characteristic of an amorphous sample, became smaller with increasing C_I_, and the intensity of the third component of the spectrum decreased as well. It is noteworthy that the R_4_ “hole” sizes, determined by the PALS method (Table 3), are comparable with the size of the crystallites determined by X-ray diffraction (Table 1) and are practically the same as micropore sizes (5.5–6.0 Å) obtained for PPO powders from CO_2_ sorption isotherms at 273 K [5]. It indirectly confirms the nanoporous structure of the crystalline phase of the PPO.

### 3.4. NMR Relaxation

The spin-lattice relaxation time T_1_ is one of the common parameters measured in TD-NMR. For solids, in most of the cases, T_1_ increases with an increase of crystallites dimensions, and, therefore, T_1_ can characterize the crystallinity.

Longitudinal or spin-lattice NMR relaxation occurs when the spin energy is dissipating to the lattice, normally contributing to a very light increase of molecular motions energy. Because of rotating or vibrating functional groups, this process is far more effective on the crystallite surfaces than in the internal areas of crystallites, where mechanical movements are usually constrained. Thus, for larger crystallites the thermodynamic equilibrium between spin system and lattice takes longer, making T_1_ higher for higher crystalline samples of similar chemical structure. This effect is observed for various materials [26,27,28,29,30].

**Figure 5:**
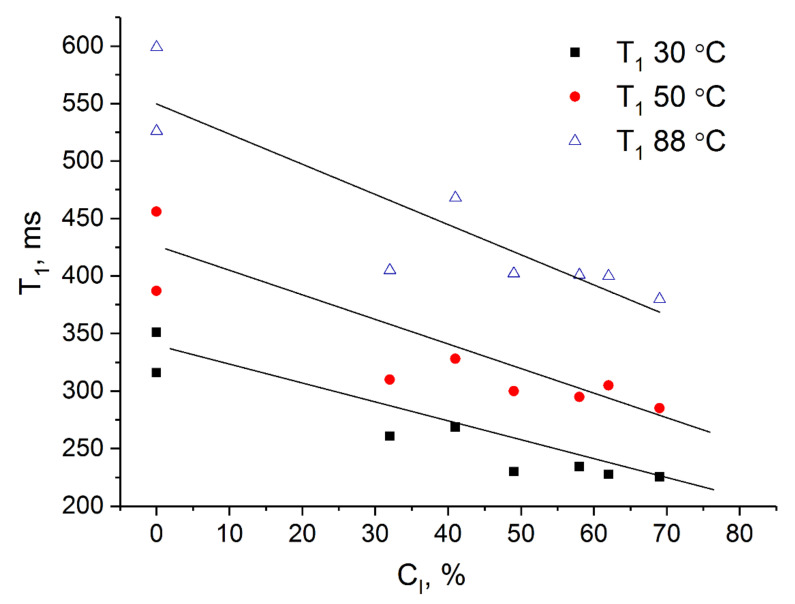
Dependence of spin-lattice relaxation time on the degree of crystallinity of PPO powders (the same samples as the ones presented previously in [5] and studied in this work) and films (this work). Points of 0% crystallinity (2 points for each temperature) represent completely amorphous PPO-2/50 °C and completely amorphous powder (the same sample as the one presented previously in [5] and studied in this work).

In the case of PPO (Figure 5), the dependence of T_1_ on crystallinity shows an inverted trend. An explanation of the phenomenon might be found, apparently, in the fact that the distance between spins in crystallites is smaller than in the amorphous phase. Consequently, the density of the crystalline phase is less than the density of the amorphous one.

Another interesting observed effect is that the spin-lattice relaxation time increases with the sample temperature (Figure 5). On one hand, T_1_ should decrease with the intensification of the rotation of the surface functional groups, but if the packing of ^1^H nuclei in crystallites turns to be less dense, the spin diffusion contribution in relaxation decreases [31], and measured T_1_ increases. So, the most likely explanation is that the average density of PPO crystallites drastically decreases with the temperature within 30–90 °C.

### 3.5. Gas Transport Parameters of PPO Films and Their Temperature Dependences

The *P*, *D*, and *S* = *P*/*D* values were obtained at 5–6 different temperatures in the range of 25–50 °C. The dependences of ln*P*, ln*D*, and ln*S* on the inverse temperature plotted in Arrhenius coordinates had correlation coefficients R^2^ in range of 0.984–0.999. The value of E*_P_* = E*_D_* + ΔH*_S_* depends on the diffusion activation energy (E*_D_* > 0) and the negative heat of sorption. Therefore, depending on these values, the apparent activation energy of permeation E*_P_* can be either positive or negative.

A decrease in the density of PPO films, and an increase in the free volume and the degree of crystallinity (Table 2), also lead to a sharp increase in the permeability coefficients for all gases (Table 4). Gas permeability data for the films are close to the ones from [5] for the samples of the respective degree of crystallinity. Gas separation selectivities for semi-crystalline films of PPO-1, PPO-2, and PPO-4 change slightly, as it was in [5].

The E*_P_* value for each of the gases, in general, decreases with an increase in the degree of crystallinity and permeability of PPO samples, although the values for PPO-1 and PPO-2 are close. Nevertheless, there is a dependence observed for E*_P_*: PPO-2/50 °C > PPO-1 ≥ PPO-2 > PPO-4. Attention is drawn to the extremely low value of E*_P_* for CO_2_ for all of PPO film samples. Moreover, if negative E*_P_* values for CO_2_ are not an exception for semi-crystalline films, given the high values of P(CO_2_) > 100 Barrer [32], the absence of a temperature dependence of CO_2_ permeability coefficient for a moderately permeable amorphous PPO-2/50 °C sample looks unexpected. High values of E _P_ for hydrogen and helium, exceeding the values of E*_P_* for oxygen, both for amorphous PPO-2/50 °C and for semi-crystalline PPO samples, are also noteworthy. This can occur due to the low heat of sorption of hydrogen and helium and is usually found in highly permeable polymers [32], for example, polyacetylenes [33]. Similar “anomalies” have also been noted for polymers belonging to the class of organic molecular sieves, for example, moderately permeable polypyrrolones [34], as well as highly permeable polymers with intrinsic microporosity (PIM-1 [35] or PIM-TMN-Trip [36]). For such polymers, this may be due to the ordering of chain packing in the amorphous phase [37] and the so–called “entropic selectivity” typical for such polymers and carbon molecular sieves [38,39,40,41]. It can be assumed that even in the X-ray amorphous PPO, there are regions with an increased ordering of the chain packing, or nuclei of the crystalline phase. This is confirmed by the data of X-ray diffraction (asymmetry of the amorphous halo and an additional peak with an angular position of 8.5 degrees) and ATR-FTIR spectroscopy (signs of the crystalline phase in the near-surface layers) of PPO-2/50 °C. For the most permeable PPO-4 sample with the highest degree of crystallinity, the E*_P_* for oxygen also tends to 0, and the E*_P_* values for all gases approach those of highly permeable polyacetylenes [32,33]. At the same time, the values of E*_P_* of oxygen, carbon dioxide, and methane, for PPO-4, are less than those for PIM-1 [35,42,43] and PIM-BTrip [36], i.e., for polymers with significantly higher permeability coefficients.

Gas diffusion coefficients, diffusion selectivities, and diffusion activation energies of the studied PPO films are provided in Table 5.

As in [5], with a decrease in the density of films, and an increase in FFV and the degree of crystallinity, gas diffusion coefficients increase for all of the studied gases. Diffusion coefficients are close to those obtained in [5] for the samples with the respective degrees of crystallinity. The selectivity of diffusion varies slightly, within the experimental error.

The E*_D_* value for all of the gases, in general, decreases with an increase in the degree of crystallinity and permeability of PPO samples, although the values for PPO-1 and PPO-2 are close. Nevertheless, there is the same dependence for E*_D_* as for E*_P_*: PPO-2/50 °C > PPO-1 ≥ PPO-2 > PPO-4. It is noteworthy that for an amorphous PPO-2/50 °C sample and for PPO-1 sample, the values of E_D_ for oxygen and CO_2_ are almost the same, and for PPO-2 and PPO-4 samples, the value of E*_D_* for CO_2_ is less than that for oxygen. This kind of pattern is again typical for organic molecular sieves class of polymers, for example, polypyrrolones [34] and polymers of intrinsic microporosity [35,36,42,43], and for some highly permeable polyacetylenes [32,33].

According to the literature data [1,44,45], the E_D_ value for polymers should linearly depend on the cross-sectional area of the gas molecule, i.e., on the square of the kinetic diameter. The scale of effective kinetic diameters d_eff_ is often used for such correlations [45] for polymers. It is specific for this scale that d_eff_ (O_2_) < d_eff_ (CO_2_) ≤ d_eff_ (N_2_). In some works, a scale of kinetic diameters for zeolites is used for such correlations [1,46], which is characterized by the following relationship: d (CO_2_) < d (O_2_) < d (N_2_). Since the E_D_ values for CO_2_ are generally less than those for oxygen in the case of PPO films, we used both kinetic diameter scales for such correlations. Determination coefficients R^2^ of linear correlations E*_D_*(d^2^) are presented in Table 6.

As can be seen from Table 6, in general, both scales are satisfactorily applicable for amorphous PPO-2/50 °C. At the same time, the correlation E*_D_* (d^2^) for PPO-1 obtained using the scale of effective kinetic diameters for polymers [45] is significantly better than that obtained using the kinetic diameters scale for zeolites [46]. The scale of kinetic diameters for zeolites [46] fits much better for E*_D_* (d^2^) correlations for PPO-2 and PPO-4 samples. This is not typical for polymer materials, in general [1]. Thus, the nanoporosity of semi-crystalline PPO film samples is also manifested in E_D_ correlations versus the cross-sectional area of the gas molecule, more typical for zeolites rather than polymers.

The gas solubility coefficients calculated as *S* = *P*/*D* and the heat of gas sorption values for the studied PPO film samples are presented in Table 7.

The gas solubility coefficients for all polymer samples increase with an increase in the critical temperature of gases [1]. As it has been previously seen in [5], the transition from amorphous PPO-2/50 °C to semi-crystalline PPO-1, PPO-2, and PPO-4 samples is accompanied by significant increase in solubility coefficients of all gases. However, in contrast to the data provided in [5], when the degree of crystallinity is 50–70% (Table 7), the increase in the degree of crystallinity and free volume has little effect on solubility coefficient. It should be noted that sorption isotherms of CO_2_ and CH_4_ were previously obtained in [7,8] at 30 °C for the amorphous PPO sample, as well as for semi-crystalline samples of PPO with a predominant β–modification (cast from benzene) and α–modification of the crystalline phase (cast from CCl_4_). The solubility coefficients of these samples can be estimated using the aforementioned isotherms and compared with the data obtained in present work. In [7,8], the solubility coefficients of CH_4_ and CO_2_ for amorphous PPO are 26 × 10^−3^ and 79 × 10^−3^ cm^3^ (STP)/(cm^3^·cmHg), respectively, which correspond (within the measurement error) to the data in Table 7. The solubility coefficients of CH_4_ and CO_2_ for β–modification of the crystalline phase are 55 × 10^−3^ and 117 × 10^−3^ cm^3^ (STP)/(cm^3^·cmHg), respectively [7,8], which also corresponds to the data in Table 7 for PPO-1, PPO-2, and PPO-4 samples. The solubility coefficients of CH_4_ and CO_2_ for α–modification of the crystalline phase are 43 × 10^−3^ and 99 × 10^−3^ cm^3^ (STP)/(cm^3^·cmHg), respectively [7,8], which is slightly lower than *S* for the β-modification and also slightly lower than the data for the PPO-4 sample (Table 7), in which the XRD method reveals the α-modification of the crystalline phase. It is possible that an increase in the proportion of α-modification in PPO-4 sample leads to an increase in the degree of crystallinity and a decrease in the solubility coefficients, which, in general, is reflected in the weak dependence of *S* on the degree of crystallinity in semi-crystalline PPO-1, PPO-2, and PPO-4 samples.

The ΔH_S_ values for all PPO samples change little with an increase in the degree of crystallinity (Table 7). In general, replacing an amorphous PPO-2/50 °C sample for semi-crystalline PPO-2 and PPO-1 leads to a slight increase in ΔH_S_ for all gases. However, for PPO-4 sample with the highest degree of crystallinity, the value of ΔH_S_ decreases almost to the level of that of the amorphous PPO-2/50 °C sample. It can be explained by the presence of an α-modification of the crystalline phase with solubility coefficients lower than that of the β-modification according to the data of [7,8]. At the same time, ΔH_S_ values for oxygen and nitrogen are practically the same within the measurement error for all of PPO samples. The heat of sorption is slightly greater than the value of E*_D_* for CO_2_ (Table 5), which leads to zero or negative values of E*_P_* (Table 4).

**Figure 6:**
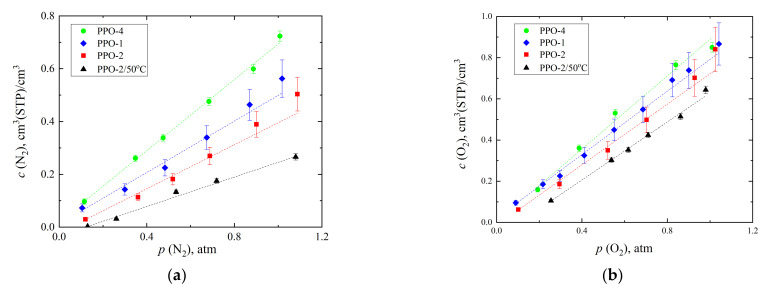

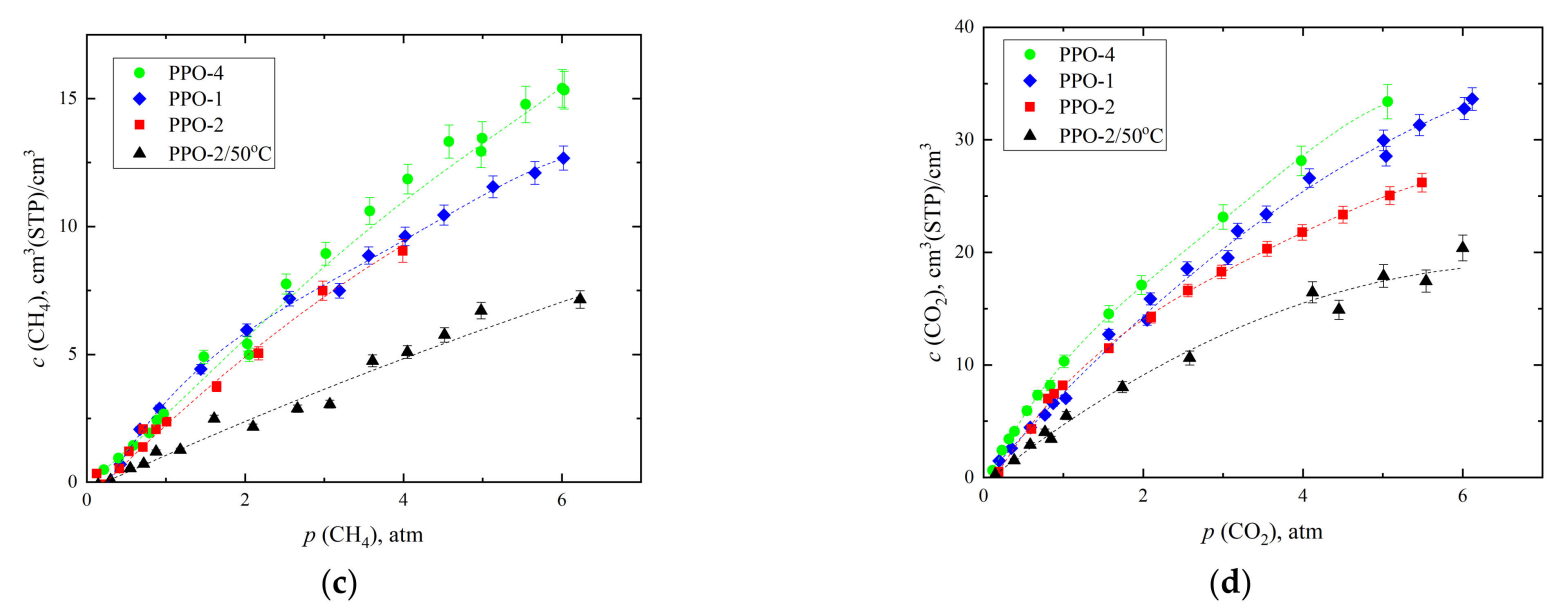
Sorption isotherms of N_2_ (**a**), O_2_ (**b**), CH_4_ (**c**), and CO_2_ (**d**) obtained by the volumetric method with chromatographic detection for PPO-2/50 °C, PPO-1, PPO-2, and PPO-4 film samples.

The sorption isotherms of O_2_, N_2_, CO_2,_ and CH_4_ for the studied PPO film samples were obtained by volumetric method with chromatographic detection [20] at 35 °C. These isotherms are shown in Figure 6.

As can be seen from Figure 6, the sorption isotherms of N_2_ (Figure 6a) and O_2_ (Figure 6b) are linear, while for isotherms of CH_4_ (Figure 6c), at least for PPO-1 and PPO-4 samples deviations from linearity are observed. Finally, deviations from linearity are obvious for all PPO samples, both amorphous (PPO-2/50 °C) and semi-crystalline (PPO-1, PPO-2, and PPO-4), for CO_2_ sorption isotherms (Figure 6d). A similar situation was observed for CO_2_ and CH_4_ sorption isotherms in [7,8]; however, in these works the nonlinearity was less pronounced since the isotherms were obtained at the pressure in the range from 0 to 1 atm. Moreover, according to [7,8], the entire range of studied pressures *S* of CO_2_ and CH_4_ changes in the following series: amorphous PPO < PPO (α-modification) < PPO (β-modification). Isotherms shown in Figure 6 demonstrate that *S* changes in the following order for all gases: PPO-2/50 °C < PPO-2 < PPO-1 < PPO-4, i.e., exactly in the order of the degree of crystallinity increase (Table 7), despite the signs of α-modification in the PPO-4 sample provided by XRD method.

A comparison of the experimental solubility coefficients obtained by various methods at 35 °C is presented in Table 8.

As can be seen from Table 8, the coefficients of solubility in PPO samples obtained by various methods do not differ from each other within the measurement error, and, in general, coincide for a similar level of crystallinity (in [5] PPO-4 sample had a higher degree of crystallinity and PPO-1 and PPO-2 samples had a lower ones). The greatest differences between the volumetric method data and the indirect method (*S* = *P*/*D*) data are observed for PPO-4, which demonstrates signs of α-modification of the crystalline phase according to the XRD data. Apparently, solubility coefficient values depend only on the free volume, and, consequently, on the degree of crystallinity of the PPO sample. The similarity of the *S* values obtained by the volumetric method and by the indirect method shows that the data of indirect calculations (*S* = *P*/D) and, consequently, the values of ΔH_S_ obtained from them are close to an equilibrium state.

Overall, the analysis of PPO transport parameters and their temperature dependences shows that all PPO samples, including amorphous PPO, differ from “conventional” amorphous and semi-crystalline polymers. By its transport properties, PPO can be attributed to the class of organic molecular sieves [34,35,36,42,43]. Apparently, this feature, as well as the ability to form a low-density crystalline phase, is associated with the regular structure of polymer chains, due to the synthesis method [4]. Changes in transport characteristics directly depend on the degree of crystallinity: with an increase in the degree of crystallinity and free volume, *P*, *D*, and *S* increase, and the values of E*_P_* and E*_D_* decrease to the level typical for highly permeable polymers. At the same time, the dependence of transport parameters on the presence of various polymorphic α- or β-modifications of the nanoporous crystalline phase is not obvious based on these results.

The similarity of ΔH_S_ values for an amorphous PPO-2/50 °C and semi-crystalline PPO-1, PPO-2, and PPO-4 samples (Table 7) deserves a separate discussion. Sorption in nanoporous semi-crystalline PPO samples should occur both in the amorphous and in the crystalline phase. In two-phase systems, the value of ΔH_S_ is additively related to the fraction of each of the phases. Then, with an increase in the degree of crystallinity, the proportion of sorbed gas molecules in the amorphous phase decreases and in the crystalline phase increases; therefore, the contribution of the amorphous phase to the total value of ΔH_S_ decreases, and the contribution of the crystalline phase increases. From the fact that ΔH_S_ values obtained from temperature dependences are close to an equilibrium state, and from the similarity of ΔH_S_ values for an amorphous PPO-2/50 °C sample and semi-crystalline PPO-1, PPO-2, and PPO-4 samples (Table 7), it follows that the values of ΔH_S_ in the amorphous and nanoporous crystalline phases do not differ significantly. Confirmation and explanation of this unexpected observation requires further research.

## 4. Conclusions

The physicochemical and gas separation characteristics study of PPO film samples with different degrees of crystallinity (from 0 to 69%) confirms the nanoporosity of the permeable crystalline phase. According to FTIR spectroscopy data, semi-crystalline films are heterogeneous: their phase composition differs in the depth of the film and near the surface. The average size of “holes” or nanopores in samples was determined by the method of positron annihilation and was close to the size of crystallites determined by X-ray diffraction. The NMR relaxation method shows that the density of the amorphous phase is significantly higher than that of the crystalline phase; with an increase in temperature, the densities of both the amorphous and the crystalline phases decrease. The activation energy of permeation of CO_2_ for amorphous PPO is close to zero, and for semi-crystalline samples it is negative since the heat of sorption exceeds the activation energy of diffusion. At the same time, the activation energies of permeation are unusually high for hydrogen and helium. The activation energies of permeation and diffusion for all gases decrease with an increase in the degree of crystallinity, while the heat of sorption is virtually independent of the degree of crystallinity. In essence, in regard to its transport properties, PPO can be included in the class of organic molecular sieves.

## Figures and Tables

**Table 1:** Angular positions of diffraction peaks, their corresponding d-spaces (*d_hkl_*), crystallite sizes (*D_hkl_*), relative peak intensities (*I_hkl_*), and crystallinity degrees (C_I_), for PPO-1, PPO-2, and PPO-4 films.

Sample	PPO-1	PPO-2	PPO-4
*hkl*	2*θ*, deg		
100	4.97	4.98	4.24
010	7.65	7.54	7.71
210	12.79	12.63	12.70
001	16.63	17.22	16.64
301	21.79	21.77	21.66
	*d_hkl_*, Å		
100	17.79	17.75	20.84
010	11.55	11.73	11.47
210	6.92	7.01	6.97
001	5.33	5.15	5.33
301	3.46	3.37	4.10
	*D_hkl_*, nm		
100	5.0	5.1	5.2
010	4.1	4.1	4.8
210	3.2	3.3	3.3
001	2.1	2.8	1.8
301	1.9	2.0	1.8
	*I_hkl_*, %		
100	5.6	3.7	7.0
0.10	12.4	14.2	9.0
210	20.3	22.1	18.6
001	11.6	5.0	17.9
301	12.5	12.9	16.6
C_I_, %	62.4	57.9	69.2
Unit cell volume, Å^3^	1095.6	1072.3	1272.5

**Table 2:** The degree of crystallinity (C_I_), density (p), and fractional free volume (FFV) of the studied PPO film samples.

Sample	*M_w_*·10^−3^, Da	C_I_, %	ρ, g/cm^3^	FFV, %
PPO-2/50 °C	355	0	1.072	17.9
PPO-2	355	57.9	1.064	18.5
PPO-1	636	62.4	1.044	20.0
PPO-4	610	69.2	1.031	21.0

**Table 3:** Parameters of spectra of ortho-positronium lifetime in samples (PALS method).

Sample	C_I_, %	τ_3_, ns	R_3_, Å	I_3_, %	τ_4_, ns	R_4_, Å	I_4_, %
PPO-2/50 °C	0	2.50 ± 0.02	3.28 ± 0.01	27.78 ± 0.10	–	–	–
PPO-2	57.9	2.42 ± 0.11	3.21 ± 0.09	20.35 ± 1.3	5.30 ± 0.33	4.89 ± 0.15	9.87 ± 1.47
PPO-1	62.4	2.17 ± 0.07	3.01 ± 0.06	12.43 ± 0.38	6.10 ± 0.14	5.23 ± 0.06	10.67 ± 0.48
PPO-4	69.2	1.85 ± 0.09	2.71 ± 0.09	8.74 ± 0.28	6.06 ± 0.07	5.22 ± 0.03	18.29 ± 0.39

**Table 4:** Gas permeability coefficients, gas separation selectivities, and activation energies of permeation of the studied PPO films.

Sample	PPO-2/50 °C	PPO-2	PPO-1	PPO-4
FFV, %	17.9	18.5	20.0	21.0
C_I_, %	0	57.9	62.4	69.2
**Gas**	P, Barrer * (30 °C)			
He	63 ± 3	101 ± 5	111 ± 6	153 ± 7
H_2_	91 ± 5	168 ± 8	188 ± 9	268 ± 13
O_2_	12 ± 1	30 ± 1	35 ± 2	53 ± 3
N_2_	2.5 ± 0.1	7.1 ± 0.4	9.0 ± 0.4	14.0 ± 0.7
CO_2_	56 ± 3	138 ± 7	176 ± 9	269 ± 13
CH4	3.2 ± 0.2	10.2 ± 0.5	13.5 ± 0.7	21.0 ± 1.0
**Gas pairs**	**Ideal selectivity**			
O_2_/N_2_	4.9 ± 0.5	4.2 ± 0.4	3.9 ± 0.4	3.8 ± 0.4
CO_2_/CH_4_	18 ± 2	14 ± 1	13 ± 1	13 ± 1
CO_2_/N_2_	22 ± 2	19 ± 2	20 ± 2	19 ± 2
He/N_2_	25 ± 3	14 ± 1	12 ± 1	11 ± 1
H_2_/N_2_	37 ± 4	24 ± 2	21 ± 2	19 ± 2
H_2_/CH_4_	29 ± 3	16 ± 2	14 ± 1	13 ± 1
**Gas**	**E*_P_*, kJ/mol**			
He	10.1 ± 0.3	6.7 ± 0.1	7.2 ± 0.2	5.4 ± 0.1
H_2_	8.5 ± 0.2	4.7 ± 0.1	5.8 ± 0.2	3.0 ± 0.2
O_2_	7.3 ± 0.2	3.0 ± 0.1	4.2 ± 0.3	0.7 ± 0.2
N_2_	12.5 ± 0.4	6.8 ± 0.2	8.0 ± 0.2	4.9 ± 0.7
CO_2_	−0.1 ± 0.1	−3.2 ± 0.1	-3.1 ± 0.3	−6.9 ± 0.3
CH4	12.6 ± 1.0	7.8 ± 0.3	8.1 ± 0.5	3.7 ± 0.4

*—1 Barrer = 10^−10^ cm^3^(STP)·cm/(cm^2^·s·cmHg).

**Table 5:** Gas diffusion coefficients, diffusion selectivities, and diffusion activation energies of the studied PPO films.

Sample	PPO-2/50 °C	PPO-2	PPO-1	PPO-4
FFV, %	17.9	18.5	20.0	21.0
C_I_, %	0	57.9	62.4	69.2
**Gas**	***D*·10^8^, cm^2^/s (30 °C)**			
O_2_	12 ± 1	18 ± 2	22 ± 2	31 ± 3
N_2_	3.6 ± 0.4	5.5 ± 0.6	7.5 ± 0.8	10 ± 1
CO_2_	6.1 ± 0.6	11 ± 1	13 ± 1	20 ± 2
CH_4_	0.96 ± 0.1	1.8 ± 0.2	2.6 ± 0.3	3.7 ± 0.4
**Gas pairs**	***α^D^* = *D_i_/D_j_***			
O_2_/N_2_	3.3 ± 0.7	3.3 ± 0.7	2.9 ± 0.6	3.1 ± 0.6
CO_2_/CH_4_	6.4 ± 1.3	6.1 ± 1.2	5.0 ± 1.0	5.4 ± 1.1
**Gas**	**E*_D_*_,_ kJ/mol**			
O_2_	21.3 ± 1.0	19.2 ± 0.7	21.0 ± 0.5	15.6 ± 0.2
N_2_	26.8 ± 3.0	23.8 ± 0.9	24.5 ± 1.0	19.5 ± 0.3
CO_2_	22.8 ± 1.4	18.1 ± 0.5	23.3 ± 1.2	14.7 ± 0.6
CH_4_	31.3 ± 1.4	27.4 ± 0.7	28.5 ± 0.6	22.7 ± 0.4

**Table 6:** Determination coefficients R^2^ of linear correlations E*_D_* (d^2^) for two types of kinetic diameter scales: for polymers (Pol) [45] and for zeolites (Zeol) [46].

Sample	PPO-2/50 °C	PPO-2	PPO-1	PPO-4
FFV, %	17.9	18.5	20.0	21.0
C_I_, %	0	57.9	62.4	68.1
*R*^2^ (Pol)	0.889	0.684	0.979	0.692
*R*^2^ (Zeol)	0.821	0.966	0.643	0.963

**Table 7:** Solubility coefficients (*S* = *P*/*D*) and heat of sorption values of gases for the studied PPO films.

	Sample	PPO-2/50 °C	PPO-2	PPO-1	PPO-4
	FFV, %	17.9	18.5	20.0	21.0
	C_I_, %	0	57.9	62.4	69.2
T_c_ [47]	**Gas**	***S*·10^3^, cm^3^(STP)/(cm^3^·cmHg) (30 °C)**			
126.2	N_2_	7.0 ± 1.0	13 ± 2	12 ± 2	14 ± 2
154.6	O_2_	10 ± 1	16 ± 3	16 ± 2	17 ± 3
190.6	CH_4_	33 ± 5	56 ± 8	52 ± 8	57 ± 9
304.2	CO_2_	91 ± 13	130 ± 19	135 ± 20	135 ± 20
T_c_ [47]	**Gas**	**−ΔH_S_, kJ/mol**			
126.2	N_2_	14.3 ± 2.7	17.1 ± 0.6	16.3 ± 0.7	15.4 ± 0.4
154.6	O_2_	14.0 ± 1.0	16.3 ± 0.5	16.7 ± 0.3	14.9 ± 0.4
190.6	CH_4_	18.8 ± 2.4	19.8 ± 0.8	20.6 ± 0.9	18.8 ± 0.4
304.2	CO_2_	22.9 ± 2.4	21.5 ± 0.7	24.8 ± 1.0	21.7 ± 0.4

**Table 8:** Comparison of gas solubility coefficients (*S*·10^3^, cm^3^ (STP)/(cm^3^·cmHg)) obtained at 35 °C by a volumetric method with chromatographic detection (desorption is highlighted in bold) and calculated as *S* = *P*/*D* in present paper and in [5] for PPO-2/50 °C, PPO-1, PPO-2, and PPO-4 film samples. The series of gases is given in the order of increasing T_c_ (Table 7); the series of PPO samples is given in the order of increasing the degree of crystallinity and free volume (Table 7).

Sample	Method	N_2_	O_2_	CH_4_	CO_2_
PPO-2/50 °C	desorption (this work)	4.4 ± 0.4	8.2 ± 0.3	22 ± 3	73 ± 13
	*S* = *P*/*D* (this work)	5.9 ± 0.9	8.6 ± 1.3	26 ± 4	82 ± 12
	S = *P*/*D* [5]	4.8 ± 0.7	7.4 ± 1.1	22 ± 3	71 ± 11
PPO-2	desorption (this work)	7 ± 1	11 ± 2	37 ± 8	87 ± 8
	*S* = *P*/*D* (this work)	11 ± 2	15 ± 2	50 ± 8	110 ± 17
	S = *P*/*D* [5]	8.6 ± 1.3	12 ± 2	39 ± 6	110 ± 17
PPO-1	desorption (this work)	8 ± 2	11 ± 2	38 ± 3	92 ± 9
	*S* = *P*/*D* (this work)	10 ± 2	15 ± 2	47 ± 7	120 ± 18
	*S* = *P*/*D* [5]	12 ± 2	15 ± 2	47 ± 7	125 ± 19
PPO-4	desorption (this work)	9.1 ± 0.4	13.0 ± 0.8	39 ± 5	105 ± 10
	*S* = *P*/*D* (this work)	12 ± 2	15 ± 2	49 ± 7	120 ± 18
	*S* = *P*/*D* [5]	15 ± 2	18 ± 3	52 ± 8	135 ± 20
